# Staged Hand-Assisted Bilateral Native Nephrectomy for Management of Posttransplant Polyuria in an Adult with Dent's Disease

**DOI:** 10.1155/2015/620371

**Published:** 2015-01-11

**Authors:** Rosa M. Montero, Rachel Hilton, Jonathon Olsburgh

**Affiliations:** Department of Nephrology and Transplantation, Guy's and St Thomas' NHS Foundation Trust, Great Maze Pond, London SE1 9RT, UK

## Abstract

Polyuria after kidney transplantation causes graft dysfunction and increased thrombotic risk. We present a case of a polyuric adult with Dent's disease who underwent staged bilateral native nephrectomies, the first operation before transplant and the second four months after transplant. This led to improved allograft function maintained during four years of follow-up. The retroperitoneal laparoscopic approach was well tolerated and allowed continuation of peritoneal dialysis before transplantation. A staged approach helps regulate fluid balance perioperatively and may be tailored to individual need according to posttransplant urine output. This novel approach should be considered for polyuric patients with tubular dysfunction including Dent's disease.

## 1. Introduction

We present a case of an adult with Dent's disease and significant polyuria who benefited from staged bilateral native nephrectomies before and after kidney transplantation using a retroperitoneal approach. Polyuria is defined as a daily urine output in excess of 3 L and is commonly seen in people with tubular dysfunction such as Dent's disease. Polyuria is also common after kidney transplantation and is due to allograft tubular dysfunction which generally resolves within the first few weeks, with the resulting functional recovery. Severe polyuria from residual native urine output combined with that of a functioning allograft may lead to difficulty in maintaining fluid balance, causing dehydration and allograft dysfunction. Native nephrectomy in polyuric paediatric patients has been shown to be beneficial [1], but it is unknown whether this is also true in adults. To our knowledge, there are no reports describing urine output following kidney transplantation in patients with Dent's disease. In addition, there is no previous description of bilateral native nephrectomies for the management of polyuria after transplantation in this patient group.

## 2. Case Presentation

A 19-year-old white male with Dent's disease developed end-stage kidney disease (ESKD) requiring peritoneal dialysis for 7 months prior to receiving a living donor kidney transplant. He was diagnosed with Dent's disease at the age of 8 years following 24-hour urinary collections with urinary calcium of 10.1 mmol/L and serum phosphate of 1.09 mmol/L with tubular reabsorption of 71%, indicating tubular phosphate loss. Plasma urate was low, 0.19 mmol/L, with urinary aminoaciduria and urinary retinal binding protein : creatinine ratio of 29999.8 indicating complete failure of proximal tubular reabsorption of low molecular weight proteins. Before transplantation, his daily urine output exceeded 3 L. After transplantation, his daily urine output increased to 4 L, resulting in great difficulty in maintaining optimal fluid balance. This subsequently led to accelerated allograft failure four years after transplantation. Kidney allograft biopsy initially showed acute tubular injury with calcineurin inhibitor toxicity; hence, ciclosporin was switched to tacrolimus. A subsequent biopsy showed moderate chronic interstitial fibrosis and tubular atrophy (Banff 97, Category 5, Grade II (a), cg0, ct2, ci2, cv1, ah1) with no acute rejection or pyelonephritis. Peritoneal dialysis was recommenced. During this period, his daily urine output averaged 3 L with 0.4 g/dL proteinuria (see Table 1).

5 years later, in order to decrease the degree of polyuria in preparation for his second kidney transplant, he underwent left laparoscopic retroperitoneal native nephrectomy, followed one month later by implantation of a kidney allograft from an unrelated living donor. The retroperitoneal approach enabled the continuation of peritoneal dialysis and the retention of the right native kidney avoided the need for severe fluid restriction in the pretransplant period.

Following the second kidney transplant, he remained polyuric with daily urine output averaging 4 L with 0.8 g/dL proteinuria (see Table 1). The immunosuppression following this kidney transplant included basiliximab as induction therapy followed by maintenance immunosuppression of prednisolone, tacrolimus, and myfortic. He required frequent hospitalisation with dehydration and significant allograft dysfunction. There was a single episode of mild T-cell mediated rejection (Banff 2009, Category 3, g0, t2, i1, v0, ah0, ptc0) which was treated with intravenous methylprednisolone. He remained polyuric, which was resistant to treatment with oral fludrocortisone (100 mcg once daily) and high dose oral desmopressin (300 mcg three times daily). His long-standing hypokalaemia with intermittent episodes of diarrhoea prevented a trial of thiazide diuretic to control his polyuria from Dent's disease.

Four months after engraftment, he underwent right laparoscopic retroperitoneal native nephrectomy without surgical complications. Both native kidneys showed histological evidence of severe chronic parenchymatous damage with calcification predominantly involving the tubulointerstitial compartment. This was thought to be a consistent primary tubular disorder such as Dent's disease. Following nephrectomy, his daily urine output decreased to 1.5 L with proteinuria decreasing to 0.25 g/dL. There was no deterioration in kidney allograft function. Four years later, serum creatinine and urine output remain stable. Urinary osmolality has normalised (see Table 1).

## 3. Discussion

Dent's disease is an X-linked recessive tubular disorder, usually due to mutations that inactivate a voltage-gated chloride transporter named as CLC-5, or less often due to mutations in the OCRL1 gene, also mutated in the oculocerebrorenal syndrome of Lowe. The disease is characterised by proximal tubular dysfunction leading to aminoaciduria, hypercalciuria, glycosuria, hypouricaemia, and low molecular weight proteinuria. Patients with Dent's disease commonly present with proteinuria, haematuria, nephrocalcinosis, and urolithiasis. Other complications include osteomalacia, rickets that may be accompanied by short stature, muscular weakness, and polyuria. Up to two-thirds of males develop some form of chronic kidney disease, usually between the ages of thirty and forty years. In contrast, females rarely develop chronic kidney disease [2]. The cause of kidney failure is unknown.

Achieving optimal hydration after kidney transplantation may be difficult if patients are severely polyuric, and this may compromise allograft function. Severely polyuric patients may also be at increased risk of transplant renal artery and/or vein thrombosis [3]. However, there may be additional contributory factors beyond polyuria itself, as patients with polyuria secondary to cystinosis do not have worse graft outcomes [4] nor have worse graft outcomes been previously reported in patients with Dent's disease. Persistent polyuria in this patient led to significant difficulty maintaining fluid balance following transplantation, resulting in accelerated loss of his first allograft and threatening his second allograft.

Polyuria has many aetiologies (see Table 2) and it is crucial to determine the precise cause, not only in order to guide therapy, but also to enable more accurate prediction of the likelihood of polyuria persisting after kidney transplantation. Polyuria due to central or neurogenic diabetes insipidus responds to intranasal or oral desmopressin. Polyuria due to tubular dysfunction is generally refractory to this approach so that maintenance of fluid balance presents a persistent challenge. Patients with an inherited cause of polyuria, such as Dent's disease, are likely to be desmopressin resistant and in such cases polyuria is likely to be exacerbated in the presence of a functioning kidney allograft. Thiazide diuretics may be beneficial treatment for polyuria arising from Dent's disease [2]; however, their use is limited in those that are hypokalaemic as in this case.

Native nephrectomy before transplantation should be considered in such patients. The benefits of bilateral native nephrectomy have previously been described in children [5, 6] undergoing surgery following kidney transplantation [7]. However, a staged approach allows those established on dialysis to maintain a reasonable fluid intake while reducing the likelihood of overwhelming polyuria immediately following transplantation. Daily urine output after transplantation will inform the decision about the requirement for the second and final nephrectomy and further demonstrates the benefits of a staged approach.

Previous reports describe a progressive decline in urine output following single native nephrectomy such that the urine output initially decreases by one-third then slowly continues to decrease over time [1]. The combination of hypoperfusion and volume depletion due to polyuria may potentiate calcineurin inhibitor nephrotoxicity [8], emphasising the importance of controlling fluid balance after transplantation. Bilateral native nephrectomies have been shown to normalise serum albumin, protein, and fibrinogen in humans [1]. Furthermore, bilateral nephrectomies for nephrotic syndrome in children may decrease the risk of graft thrombosis and intravascular depletion, while improving nutrition and wound healing [9]. Significant improvements in serum albumin, protein, and fibrinogen concentrations have been reported following single native nephrectomy in the paediatric population, and we saw similar improvements in this adult case, although a larger study would determine whether this effect is consistently observed.

Bilateral nephrectomies are standardly performed using a simultaneous transperitoneal approach, but this prevents the initiation or continuation of peritoneal dialysis [10]. This may adversely affect quality of life, firstly because peritoneal dialysis is a home-based therapy, but more particularly due to the need for the newly anephric patient to maintain a severely restricted fluid intake. Without proper patient education and dietary compliance, there is a risk of fluid overload which may be life-threatening. By contrast, a staged approach using laparoscopic retroperitoneal nephrectomy may be preferred, particularly where there is a planned living donor kidney transplant or prior to listing for a deceased donor transplant. This allows a less stringent fluid restriction on dialysis with associated improvement in quality of life. Furthermore, this minimally invasive approach results in a shorter hospital stay and reduced pain when compared with an open approach.

This case demonstrates that staged bilateral native nephrectomies were safe and well tolerated in an adult patient with Dent's disease and significant polyuria and enabled optimisation of fluid balance in the perioperative period and subsequent maintenance of stable allograft function.

## Figures and Tables

**Table 1:** Serum and urinary changes before and after nephrectomies.

	Urine output (L/day)	Proteinuria (g/dL)	Na+ (mmol/L)	K+ (mmol/L)	Serum creatinine (*µ*mol/L)	Serum albumin (g/L)	Urinary osmolarity (mOsm)
Prenative nephrectomy 11/2010	3	0.4	139	3.1	668	43	—

After 1st native nephrectomy 12/2010	3	0.6	136	3.2	831	39	—

After 2nd renal transplant 04/2011	4	0.8	141	3.8	207	40	313

After 2nd native nephrectomy 07/2011	1.5	0.25	144	4.7	167	43	301

4 years later 09/2014	1.5	0.13	139	4.8	168	49	426

**Table 2:** Causes of polyuria.

Renal tubular disorders	Nephrogenic diabetes insipidus Inherited disorders: *Dent's disease, Bartter's syndrome* Acquired disorders secondary to *multiple myeloma, sarcoidosis, hypercalcaemia, analgesic nephropathy, obstructive uropathy* Polyuric phase of recovering acute kidney injury

Endocrine disorders	Cranial diabetes insipidus Addison's disease Hyporeninemic hypoaldosteronism

Drugs	Tubular dysfunction: *diuretics, aminoglycosides, *and*lithium* Mannitol Anticholinergics Contrast agents Clonidine

Psychogenic	

## Abbreviations

L: Litres
g/dL: Grams per decilitre
mcg: Micrograms
ESKD: End-stage kidney disease.
